# Optical Microscopy Observations and Construction of Dupin Cyclides at the Isotropic/Smectic A Phase Transition

**DOI:** 10.3390/ma14164539

**Published:** 2021-08-12

**Authors:** Mikhael Halaby Macary, Gauthier Damême, Antoine Gibek, Valentin Dubuffet, Benoît Dupuy, Justin Picart, Roll Freddy Dimeni, Claire Meyer

**Affiliations:** Laboratoire de Physique des Systèmes Complexes, Université de Picardie Jules Verne, 80039 Amiens, France; mikhael.macary@gmail.com (M.H.M.); gauthierdameme@gmail.com (G.D.); antoine.gibek@gmail.com (A.G.); valentin.dubuffet@gmail.com (V.D.); benoit.dupuy24@gmail.com (B.D.); justin.picart0712@gmail.com (J.P.); dimeniroll@gmail.com (R.F.D.)

**Keywords:** bâtonnets, focal conical domains, Smectic A phase, Dupin cyclides

## Abstract

In this work, we are interested in the nucleation of bâtonnets at the Isotropic/Smectic A phase transition of 10CB liquid crystal. Very often, these bâtonnets are decorated with a large number of focal conics. We present here an example of a bâtonnet obtained by optical crossed polarized microscopy in a frequently observed particular area of the sample. This bâtonnet presents bulges and one of them consists of a tessellation of ellipses. These ellipses are two by two tangent, one to each other, and their confocal hyperbolas merge at the apex of the bâtonnet. We propose a numerical simulation with Python software to reproduce this tiling of ellipses as well as the shape of the smectic layers taking the well-known shape of Dupin cyclides within this particular bâtonnet area.

## 1. Introduction

**Figure 1:**
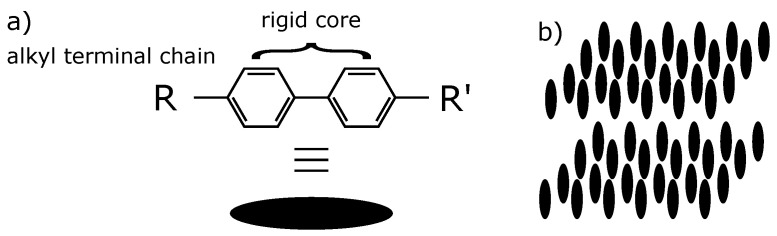
(**a**) Rod shape mesogen calamitic liquid crystal; (**b**) 3D Smectic A phase.

Thermotropic Liquid Crystals (LCs) are intermediate phases between crystalline solids and isotropic liquids, and they can be made of elongated molecules—called “mesogens”—which in turn possess a rigid core consisting of a set of phenyl groups and flexible tails of varying lengths consisting of alkyl terminal chains R and R’ as shown in Figure 1a for instance. They exhibit a very rich polymorphism—or mesomorph phases—depending on temperature: nematics, smectics A, B, C, E, etc. [1]. In a Smectic A phase (Figure 1b), the mesogens are arranged in layers with their centers of gravity distributed randomly in the plane of the layers and their axes parallel to the normal to the layers (called director). It is a sort of two-dimensional liquid in the plane of the layers and a solid in the direction of the director.

During the cooling stage from the isotropic phase to the Smectic A phase, elongated defects of different shapes appear. These defects, first reported by Friedel [2], are called bâtonnets, which is French-origin word for rods mainly used in the LC defect studies. They have been reported in different lamellar phases of new liquid crystal compounds [3,4,5,6,7]. They can be either cylindrical or spherical with rounded ends. Often, these bâtonnets exist with different types of shapes and curvatures; they can be adorned with bulges that give them the shape of balusters. These bulges are formed by a necklace of small pearls, small protrusions perfectly equal to each other, which correspond to a festoon of *focal domains* when their size is large enough, and will be developed afterwards. These domains satisfy the *law of focal conics* (see Section 2.2).

Note that these kinds of bâtonnets also exist at the Isotropic—N* phase transition and that smectic-like bâtonnets have been recently reported in N/N${}_{TB}$ biphasic samples [8,9] and in synthesized LCs from both inorganic and metal-organic precursors [10,11,12].

The growth dynamics of a bâtonnet has been investigated using *universal scaling laws of nucleus growth*. A shape anisotropy of the bâtonnet has been measured both for the length and for the width of the bâtonnet and a variation in square root of time has been reported [13]. Previous studies involving the shape of a bâtonnet have also been done and the complex structure inside has been resolved in several cases either for thermotropic [14] or lyotropic [15] LCs. The LC droplets made by the lamellar phase inside a sponge phase matrix are similar to Friedel’s bâtonnets but much simpler, and they are the result of a competition between inter-facial tension, smectic elasticity, and the growth mechanism [16]. The effect of external constraints like, for instance, the application of an electric field has also been reported (see [14,17]). The influence of anchoring energy on the prolate shape of tactoids in lyotropic inorganic LC has also been developed [18].

In this article, we will focus on the analysis of the internal structure of a particular area inside a bâtonnet in a usual thermotropic liquid crystal (10CB). We will indeed see a particular example of a bâtonnet, which is made up of a very organized set of focal conics that we will first describe in the next paragraph.

Under crossed polarizors optical microscopy, the SmA phase shows the macroscopic texture of *defects*. These defects can be either made by Focal Conic Domains FCDs [19], parts of FCD (DHs [20,21]) or FCD assemblies (oily streaks [22,23], polygonal texture [2,24], and flower texture [25]), whose simulation was recently made in a hybrid-aligned smectic with curved interfaces [26]. FCDs can be arranged in a regular lattice and might be used for example as templates for lithographic applications [27]. Bahr et al. were also interested in the manipulation of size and location of FCDs, by controlling the generation and arrangement of these latter through chemical and topographical patterning of the substrate or by varying the magnitude of anchoring strength on the substrate [28,29,30,31]. Honglawan et al. showed epitaxial assembly of the FCD lattice with a tailored domain size and symmetry using polymer based micro-pillar arrays, where they can favor the “pinning” of FCD centers near pillar edges [32,33]. These defects are present in different technological fields: going from the fabrication of functional surfaces, the self-assembly of soft micro-systems, template lithographic patterns [27], enhanced charge transport in photovoltaic cells and transistors to optical vortex generators [34]. In order to enhance their efficiency and their use in these technologies, a focus on the geometric aspect of these domains is needed. However, what are these defects and how are they structured?

Friedel and Grandjean [35] have denoted by LC the compounds mentioned by O. Lehmann [36]. They have made a precise classification of different phases and in particular nematic and smectic phases. In smectic phases, they have obtained a large scale of defects, due to the deformation of the lamellar structure. These defects take the shape of two pairs of conjugate conics, in general, an ellipse and a confocal hyperbola or two confocal parabolas, which are called Focal Conics FCs. These conics are located in two mutually orthogonal planes, in such a way that the apex of one will pass through the focus of the other and conversely.

**Figure 2:**
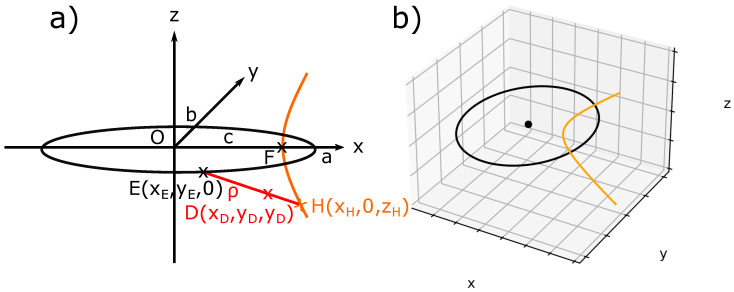
(**a**) Ellipse and hyperbola in a confocal geometry; (**b**) Python simulation of (**a**).

Let us briefly recall the FCDs geometry. Figure 2a shows an ellipse of center 0 in the (xOy) plane with semi-major axis a, semi-minor axis b, and its confocal hyperbola in the (xOz) plane. Three specific points: E(x${}_{E}$, y${}_{E}$, 0), H(x${}_{H}$, z${}_{H}$, 0), D(x${}_{D}$, y${}_{D}$, z${}_{D}$) belonging respectively to the ellipse, the hyperbola, and the Dupin cyclide are drawn. This ellipse, its center 0, and its confocal hyperbola are illustrated in Figure 2b. An FCD is defined as the domain limited by four revolutions’ cones: two cones whose apexes are the poles of the hyperbola, which lies on the ellipse and two other cones whose apexes are two diametrical points of the ellipse, which lies on the hyperbola.

It has been shown that, in order to keep their equidistant, smectic layers take the shape of *Dupin cyclides*, which are a generalization of Tori. In our study, we will take only the physical part of the FCD, which is here the one with negative Gaussian curvature (In several cases, experimental situations in SmA phase of thermotropic LC necessitate to take both negative and positive Gaussian curvature, see Figure 14 in [37] for an example). When the ellipse is degenerated into a circle and the hyperbola into a straight line, Dupin cyclides are simple parallel tori, called Toroidal Focals Conic Domains (TFCDs).

**Figure 3:**
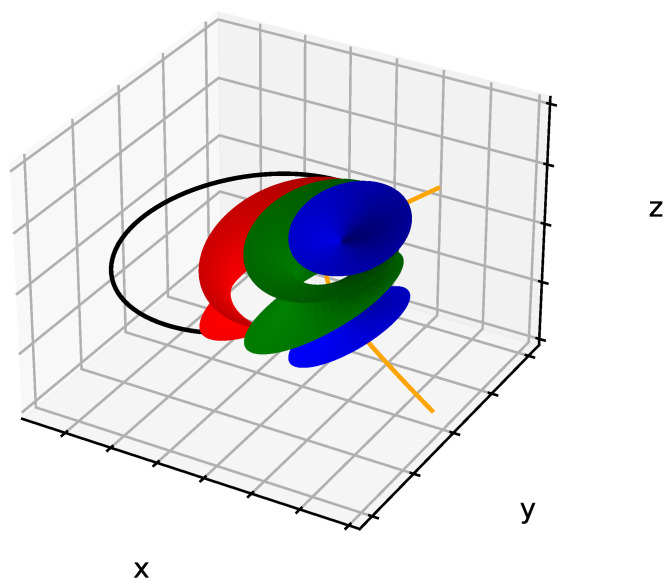
Three smectic layers corresponding to a single focal conic domain as a result of the Dupin cyclides’ equations system.

Let us consider D ($x_{D},y_{D},z_{D}$) any point between E and H belonging to a *Dupin cyclide* (Figure 2a), ρ being the distance between E and D. In order to simulate the smectic layers in the presence of FCDs, we used the parametric equations of Dupin cyclides; see Equation (1) [38]:

(1) $$\left\{\begin{array}{c} x-c\cosh v=-\frac{a\cosh v-r}{a\cosh v-c\cos u}\left(c\cosh v-a\cos u\right)\\ y-0=-\frac{a\cosh v-r}{a\cosh v-c\cos u}\left(-b\sin u\right)\\ z-b\sinh v=-\frac{a\cosh v-r}{a\cosh v-c\cos u}\left(b\sinh v\right) \end{array}\right.$$

where $c=\sqrt{a^{2}-b^{2}}$, and *u* and *v* are the parameters of the surface respectively used to describe the ellipse and the complete hyperbola (0≤u≤2π, −∞≤v≤+∞). There is only a physical part of the hyperbola, which corresponds to the two physical poles (−π/2≤v≤+π/2). Each value of r defines a single smectic layer, i.e., a single Dupin cyclide. A sample of 75 μm includes approximately 20,000 layers; nevertheless, only three of them have been illustrated in Figure 3 with the associated FC. In order to draw only the physical part of the cyclides with negative Gaussian curvature, the limits of u and v are given by:

(2) $$\begin{array}{c} u<\arccos\left(\frac{r}{c}\right)\;\;\mathrm{and}\;\;v<\mathrm{arccosh}\left(\frac{r}{a}\right) \end{array}$$

One can see using a Polarized Optical Microscope (POM) that FCDs assemble in large scale clusters, so the question is: what are the rules that they should obey? Our article will be divided as follows: first, we will present the image obtained in POM observations. Second, we will recall Friedel’s laws of association for two FCs, and in particular the law of corresponding cones. Finally, we apply these association rules to the case of two and then multiple FCs inside the selective area of our bâtonnet.

## 2. Results

### 2.1. Polarized Optical Microscopy

**Figure 4:**
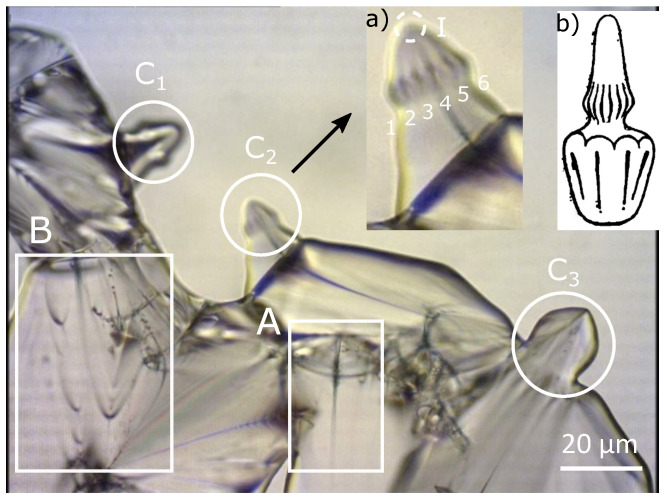
Polarized Optical Microscopy of 10CB: (**a**) Zoom in a certain region where bâtonnets are clearly visible; (**b**) bâtonnets’ model given by Friedel from [2].

In order to obtain distinctly the focal conical structure, we heat the sample of 4-*n*-decyl-4′-cyanobiphenyl (10CB) liquid crystal to the isotropic phase (52.2 °C) and then the sample has been cooled very slowly. When the temperature approaches 51 °C, the sample becomes progressively birefringence and shows the existence of a great number of objects, which move very quickly in the field of the microscope. What is interesting in this work is that a part of the smectic phase appears in the form of “*bâtonnets*” (Figure 4a,b). When temperature is further decreased, coalescence of adjacent bâtonnets can be seen and, progressively, bâtonnets will completely fill the sample and allow the place to the usual Smectic A phase. The bâtonnets, which generally have a conical structure, contain large FC when their size is large enough. The bâtonnets rotate quickly and, in order to stabilize the texture observed in Figure 4a, the temperature has been very slowly increased. When two bâtonnets of comparable sizes meet, they usually coalesce, giving rise to a bigger bâtonnet.

Looking more deeply into Figure 4, several areas are visible. Part A clearly shows: an isolated FC in the bulk, and the hyperbola seems to be degenerated into a straight line and its confocal ellipse into a circle. Figure 4 zone B presents two grain boundaries with ellipses in the observation plane, and their confocal hyperbolas are seen as part of the lines because they lie in a perpendicular plane. In Figure 4, we also see three different zones $C_{1}$, $C_{2}$, and $C_{3}$ exhibiting similar bâtonnets. In our study, we will focus on the simulation of the bâtonnet inside the $C_{2}$ zone. A zoom of this area $C_{2}$ has been illustrated in the inset (a) of Figure 4, where we observe the presence of about six FCs’ two by two tangents and decorating the bâtonnet. Due to the revolution symmetry of this bâtonnet, we assume that about six other hidden FCs exist behind them. The confocal hyperbolas are merging into the apex of the bâtonnet, which presents strong similarities with Friedel’s representation. In general, inside the bâtonnets, a variable number of FCs is present.

### 2.2. Focal Conic Assembly Corresponding to Friedel’s Law

FC assembly laws were studied by Friedel in 1922 [2], and then, in 2000, they were reviewed in a detailed way [39]. Briefly speaking, focal groups can show very wide-ranging types of arrangements, but, despite this, they are not randomly distributed and they fit together according to perfectly determined laws:

- First, the domains of two adjacent focal groups (FC) cannot overlap;
- Second, in the case where two conics are tangent at a point M, the cones of revolution with apex M and resting on the focal lengths of the conics coincide; they share a common generatrix, this is the Law of Corresponding Cones (L.C.C.).

## 3. Discussion

### 3.1. Geometrical Model

The objective of this work is to simulate the bâtonnet observed in Figure 4a. In order to do that, “roughly speaking”, we will proceed as follows:

First: Plot one set: ellipse and confocal hyperbola, which will define one single FCD.
Second: Decorate the predefined domain by adding their respective smectic layers using the Dupin cyclides’ equation system.
  *These two first steps will define one single domain (see Figure 3)*.
Third: Plot the other focal conic domains based on Friedel’s law of focal conics’ assemblies and mainly on the fact that these ellipses that have been shown in Figure 4a should be tangent to each other and cannot interfere.
  *The third step is the crucial one, which we will detail in the next paragraph, but mainly our idea is to copy the first domain and to rotate it with an angle depending on the number of focal conic domains needed*.

In the next paragraph, we will first present some graphs and equations related to FCDs, which will be very useful in order to describe our model and later we will show the results obtained for two and then for a set of multiple FCDs.

#### 3.1.1. Geometrical Definitions

**Figure 5:**
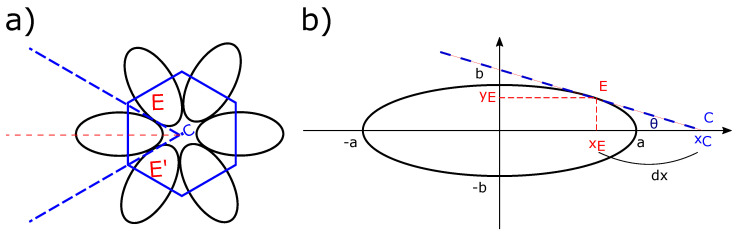
(**a**) Projection of a group of six Focal Conics in the (xOy) plane and (**b**) Zoom in on one particular ellipse; θ denotes the angle between the major axes of the ellipse and the tangent line passing through C to the ellipse.

Looking at a particular zone of Figure 4a (zone I), which corresponds to the apex of our bâtonnet, a set of two by two tangent ellipses can be seen. Figure 5a represents *one* of these patterns in the case of *six* ellipses; *other patterns will be presented afterwards*.

As we can see from Figure 5a, these ellipses are tangent and a kind of “*flower texture*” is formed, where their semi-minor axes meet in a hexagon. In order to plot tangentially a group of ellipses, our idea is as follows:

First: Plot an arbitrary FC with the corresponding smectic layers (Figure 3);
Second: Copy the graph obtained in the first step and then rotate it with a specific angle θ and with center C, which is the center of the hexagon. For this, in Python code (see Supplementary Materials), we will use a *for loop* that will enable us to plot as many ellipses as we like, and, for each loop, the FCDs and the corresponding smectic layers have been rotated using a rotational matrix. This task can be repeated as many times as needed independently from the number of FCDs contained inside the bâtonnets, see areas $C_{1}$ and $C_{2}$ in Figure 4.

*In order to use the rotational function correctly, the center C of the hexagon should be the center (0, 0, 0) of the three-dimensional axes. Therefore, we need to find the coordinate of C ($x_{C}$) to translate the FCD to the origin (0, 0, 0).*

For that, let us describe useful plots and equations, Figure 5b shows one of the six ellipses of the semi-major axis a, semi-minor axis b centered at the origin (0, 0, 0), and E ($x_{E},y_{E}$) the tangent point on the ellipse used to plot tangentially the other focal domains. The equation of the tangent of the ellipse passing through C is needed. The first derivative of the usual ellipse equation gives the needed equation:

(3) $$\frac{dy}{dx}=-\frac{b^{2}}{a^{2}}\frac{x}{y},$$

Thus, the slope equation for a tangent on E is:

(4) $$\alpha_{E}=-\frac{b^{2}x_{E}}{a^{2}y_{E}}\Rightarrow \alpha_{E}^{2}=\frac{b^{2}x_{E}^{2}}{a^{2}\left(a^{2}-x_{E}^{2}\right)},$$

Using $\alpha_{E}$, we will correlate now $x_{E}$ with θ:

(5) $$\alpha_{E}=\tan\theta =\frac{y_{E}}{x_{E}},$$

Therefore,

(6) $$\frac{a^{2}}{b^{2}}\tan^{2}\theta =\frac{x_{E}^{2}}{a^{2}-x_{E}^{2}}$$

That is, by regrouping x${}_{E}$ terms:

(7) $$x_{E}^{2}=\frac{\frac{a^{4}}{b^{2}}\tan^{2}\theta }{1+\frac{a^{2}}{b^{2}}\tan^{2}\theta },\;\mathrm{with}\;\theta =\frac{\frac{2\pi }{n}}{2}=\frac{\pi }{n},$$

After correlating $x_{E}$ with θ, our next step is to define the coordinate of $x_{C}$ in order to center our graphs by translating them of $-x_{C}$. Let us define dx as the distance between $x_{E}$ and $x_{C}$ (Figure 5b); since C belongs to the tangent line, we can write it as:

(8) $$y_{E}+\alpha_{E}dx=0\Rightarrow dx=-\frac{y_{E}}{\alpha_{E}}$$

(9) $$dx^{2}=\frac{y_{E}^{2}}{\alpha_{E}^{2}}=\frac{\left(a^{2}-x_{E}^{2}\right)\frac{b^{2}}{a^{2}}}{\frac{b^{2}}{a^{2}}\frac{x_{E}^{2}}{\left(a^{2}-x_{E}^{2}\right)}}=\frac{{\left(a^{2}-x_{E}^{2}\right)}^{2}}{x_{E}^{2}}$$

(10) $$\mathrm{Or}\;:\;x_{C}=x_{E}+dx\;\mathrm{therefore}:\;\;x_{C}=x_{E}+\frac{a^{2}-x_{E}^{2}}{x_{E}}$$

#### 3.1.2. Application on Two FCDs

**Figure 6:**
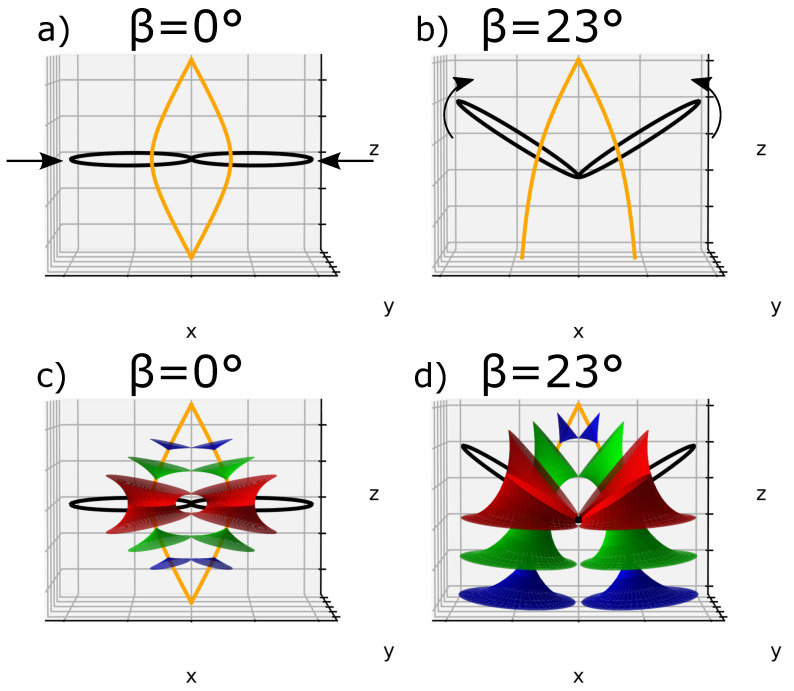
(**a**) The *flatten* case of two FCDs plot it tangentially according to Friedel’s law (l.c.c.) with their respective vertexes merging at the top. (**b**) the *tilted* case of the same FCDs with randomly tilt angle β=23°; (**c**,**d**) three equidistantly smectic layers corresponding respectively to the *flatten* and *tilted* FCDs.

**Figure 7:**
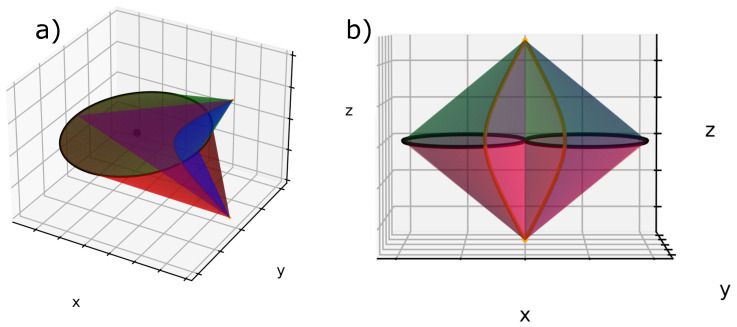
(**a**) FCD limited by four revolution cones: two of them lie on the ellipses and the two others lie on the hyperbolas; (**b**) two adjacent FCDs: their ellipses are tangent, and their confocal hyperbolas are merging at two poles. The two cones posses a common generatrix in agreement with the law of corresponding cones.

Let us consider one single ellipse and let us call β the angle between the ellipse plane and the horizontal plane (xOy). If we plot a second ellipse tangentially to the first one with their confocal hyperbolas, we obtain Figure 6a, where β=0°; this case will be called the *flatten* case. In Figure 6a, we can see two poles of hyperbolas converging at the apex of the bâtonnet similar to zone I in Figure 4a. (Figure 7a and b represent respectively the domains of one and two adjacents FCDs).

Another perspective can be considered, when the ellipses are *tilted* with respect to the horizontal plane with an angle β (β≠0°), this case will be designated by the *tilted* case. For demonstration purposes, we showed in Figure 6b a particular case where β has been randomly chosen as 23°.

Three corresponding smectic layers using Dupin cyclide equation systems in both *flatten* and *tilted* cases were added and presented in Figure 6c,d, where we can see that the layers are equally distributed and also obey the l.c.c.

**Figure 8:**
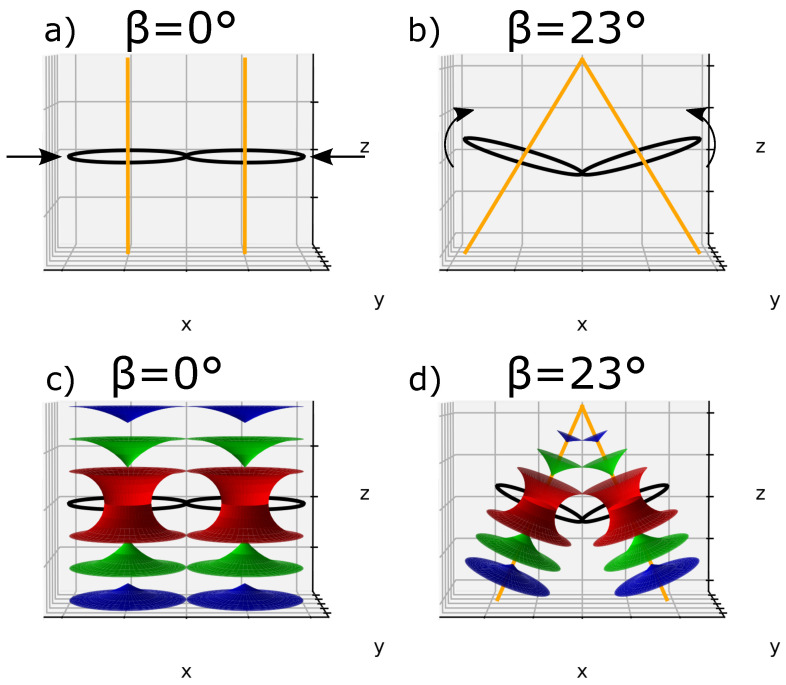
(**a**) The *flatten* case of two TFCDs whose circles are mutually tangent according to Friedel’s law (l.c.c.) with their respective parallel straight lines, (**b**) The *tilted* case of the same TFCDs with a random angle of inclination β=23°, where the straight lines are converging at the top, (**c**,**d**) three equidistantly smectic layers attributed, respectively, to the *flatten* and the *titled* cases of TFCDs.

Two TFCDs are also presented, where the ellipses and their confocal hyperbolas are respectively degenerated into a circle and a straight line. In the same way as done before, Figure 8a presents the *flatten* case of two TFCDs (β = 0°). To achieve two different lines merging at the apex of the bâtonnet (zone I in Figure 4a), circles and straight lines have been tilted with an angle β. Figure 8b represents a particular case where β has been randomly chosen to be 23°.

For both *flatten* and *tilted* cases, three smectic layers were respectively represented in Figure 8c,d, where the Tori are equally distributed in the space and follow the l.c.c.

#### 3.1.3. Application on Sets of FCDs

**Figure 9:**
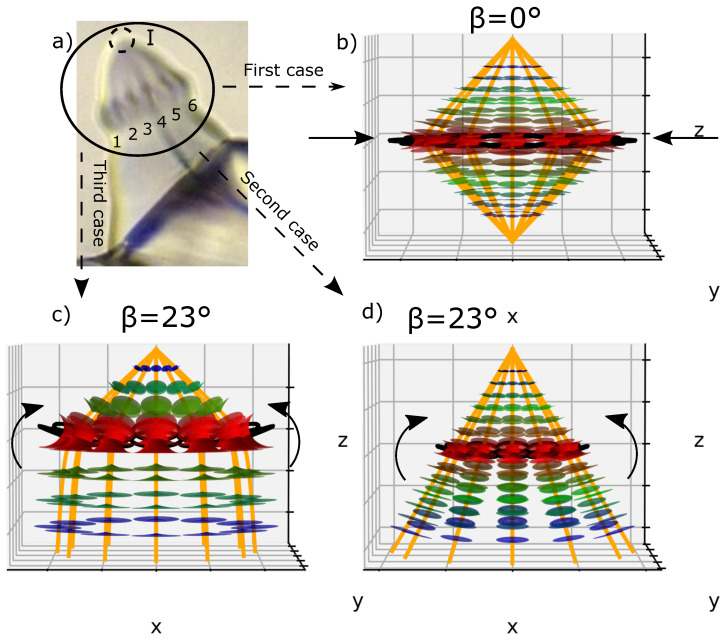
(**a**) Recall of the bâtonnet obtained in POM observation; (**b**,**c**) represent respectively the *flatten* and *tilted* cases of twelve FCDs and their respective smectic layers plotted tangentially to each other according to Friedel’s law (l.c.c.); (**d**) the *tilted* case of TFCDs with their respective smectic layers.

In the previous paragraph, we presented the simulation in a particular case of two FCDs. Nevertheless, as we can observe in the POM image (Figure 9a), approximately twelve FCs are present, six of them were enumerated—two by two tangents and their confocal hyperbolas merging into the apex of the bâtonnet. To represent these situations, we have to duplicate the task made to plot Figure 6 and Figure 8, but, in this case, with twelve iterations.

The *flatten* case of twelve FCDs with their respective 7 smectics layers according to Dupin cyclide equations’ systems are presented in Figure 9b. Figure 9c represents a particular example of the *tilted* case of 4 smectic layers where the value of β has been randomly chosen as 23°.

Finally, a particular example of the *tilted* case for twelve TFCDs with their respective 7 smectic layers, where the value of β has been randomly chosen as 23°, is presented in Figure 9d.

In all three cases, we can see that all the FCDs and TFCDs are in agreement with l.c.c., and they are tangent one to each other. Their confocal hyperbolas and straight lines were also presented and look similar to what has been obtained in a POM image—zone I, they are connected at the top of the bâtonnet.

## 4. Materials and Methods

**Figure 10:**
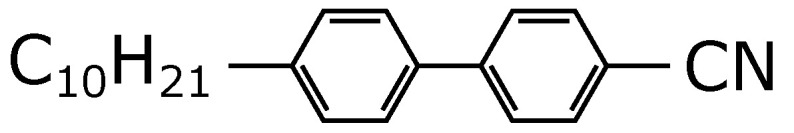
Molecule structure of 4-*n*-decyl-4′-cyanobiphenyl (10CB) liquid crystal.

Polarized Optical Microscopy (POM) was the characterization method mainly used in our study to observe the bâtonnet at the isotropic semctic A phase transition in our material. We used 4-*n*-decyl-4′-cyanobiphenyl (10CB) liquid crystal having the chemical structure of Figure 10 and the following phase transition sequence Isotropic $\overset{52.2\;{}^{\circ}C}{\to }$ SmA. The simulation of the smectic layers has been done using Python 2.8.3 Version.

## 5. Conclusions

In this work, we were interested in the understanding of the statique of a bâtonnet. We proposed a model to simulate a specific zone inside a bâtonnet of 10 CB liquid crystal sample during the isotropic-SmA phase transition. These bâtonnets contain a certain number of FCDs arranged according to specific laws and, in particular, the law of corresponding cones. The number of FCDs can vary from one bâtonnet to another, but the internal structure of the bâtonnets are fundamentally the same. Taking this into consideration, our model can be used to simulate any number of FCDs present inside a bâtonnet. Nevertheless, we just have to mention that our model assumes that the potential energy function is isotropic, and, therefore, it is only applicable to the molecules with relatively small polarity. Understanding the statics of these defects will help us to know the dynamics of formation of bâtonnets more in depth, which in turn enhances our knowledge on the morphological properties of liquid crystals for further applications in technological fields. For instance, it would be very interesting to study the coalescence of bâtonnets and the effect of an electric field in their shape change behavior.

## Data Availability

No new data were created or analyzed in this study. Data sharing is not applicable to this article.

## Supplementary Materials

The Python program used in this work is available online via GitHub at https://github.com/Mikhael-HALABY-MACARY/FCDs—and-Dupin-cyclides—tiling—inside—batonnet.git (accessed on 8 August 2021).

## Abbreviations

The following abbreviations are used in this manuscript:

LC	Liquid Crystal
FCs	Focal Conics
FCDs	Focal Conic Domains
TFCDs	Toroidal Focal Conic Domains
Smectic A	SmA
